# Occurrence, Distribution, Evolutionary Relationships, Epidemiology, and Management of Orthotospoviruses in China

**DOI:** 10.3389/fmicb.2021.686025

**Published:** 2021-08-04

**Authors:** Zhongkai Zhang, Kuanyu Zheng, Lihua Zhao, Xiaoxia Su, Xue Zheng, Tiantian Wang

**Affiliations:** Key Lab of Agricultural Biotechnology of Yunnan Province, Biotechnology and Germplasm Resources Research Institute, Yunnan Academy of Agricultural Sciences, Kunming, China

**Keywords:** *Orthotospovirus*, species diversity, systemic infection, multiple transmission routes, monitoring and early warning

## Abstract

Orthotospoviruses are responsible for serious crop losses worldwide. Orthotospoviral diseases have spread rapidly in China over the past 10 years and are now found in 19 provinces. Currently, 17 *Orthotospovirus* species have been reported in China, including eight newly identified species from this genus. The number of new highly pathogenic *Orthotospovirus* strains or species has increased, likely because of the virus species diversity, the wide range of available hosts, adaptation of the viruses to different climates, and multiple transmission routes. This review describes the distribution of *Orthotospovirus* species, host plants, typical symptoms of infection under natural conditions, the systemic infection of host plants, spatial clustering characteristics of virus particles in host cells, and the orthotospoviral infection cycle in the field. The evolutionary relationships of orthotospoviruses isolated from China and epidemiology are also discussed. In order to effectively manage orthotospoviral disease, future research needs to focus on deciphering the underlying mechanisms of systemic infection, studying complex/mixed infections involving the same or different *Orthotospovirus* species or other viruses, elucidating orthotospovirus adaptative mechanisms to multiple climate types, breeding virus-resistant plants, identifying new strains and species, developing early monitoring and early warning systems for plant infection, and studying infection transmission routes.

## Introduction

Orthotospoviruses have a worldwide distribution and cause serious economic losses in a variety of crops (Komoda et al., 2017). Before 2017, orthotospoviruses were considered to be part of the genus *Tospovirus* in the family Bunyaviridae and were divided into seven or nine serogroups based on serology (Chen et al., 2012b, 2016). However, in 2020, the International Committee on Taxonomy of Viruses (ICTV) announced that the genus *Orthotospovirus* belongs to the family Tospoviridae, order Bunyavirales, class Ellioviricetes, realm Riboviria, subphylum Polyploviricotina, phylum Negarnaviricota, and kingdom Orthornavirae (Hong et al., 2020).

As with other members of the order Bunyavirales, orthotospovirus particles are spherical, and multi-virions form aggregations in host-derived vesicles. Mature virions range in diameter from 80 to 120 nm. The surface of the virions is composed mainly of two glycoproteins (Gn and Gc), which are responsible for the virus acquisition and transmission by the thrips vectors. The core of the virion contains three viral RNA fragments, named L RNA, M RNA, and S RNAs according to their lengths, encapsulated by the nucleocapsid protein (N). The orthotospovirus are single-negative-stranded, ambisense RNA viruses. The L RNA (∼8.9 kb) encodes the RNA-dependent RNA polymerase (RdRp) in the complementary (vcRNA) strand; M RNA (∼4.8 kb) encodes the movement protein (NSm) in the viral (vRNA) strand and the Gn and Gc proteins in the vcRNA strand; and RNA S (∼2.9 kb) encodes the N protein in the vcRNA and silencing suppressor (NSs) in the vRNA strand (Oliver and Whitfield, 2016).

To date, many articles have illustrated details of the global occurrence, epidemiology, and molecular interactions between orthotospoviruses and their thrips vectors (Pappu et al., 2009; Oliver and Whitfield, 2016). In this article, we will summarize the latest research progress on species diversity, occurrence, distribution, epidemiology, and management of orthotospoviruses in China.

## Symptomatology

**FIGURE 1:**
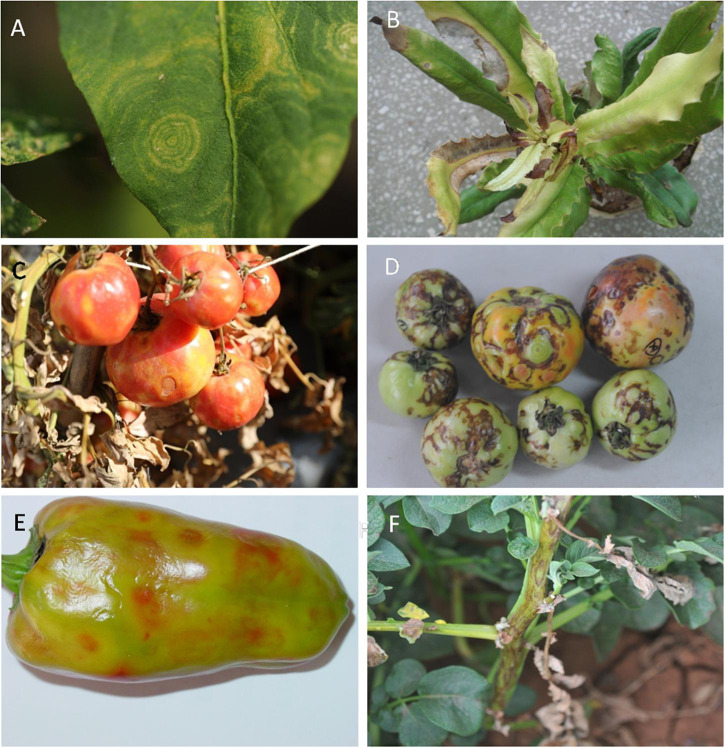
Typical symptoms of host plants infected with orthotospoviruses under natural field conditions. **(A)** Zonate spot in leaf of chili pepper (*Capsicum annuum* L.) infected with tomato zonate spot orthotospovirus (TZSV). **(B)** Necrotic spot in leaf of macadamia nut (*Macadamia ternifolia* F. Muell.) infected with *watermelon silver mottle virus* (WSMoV) serogroup member. **(C)** Yellow spot and ringspot in tomato (*Solanum lycopersicum* L.) infected with *tomato spotted wilt orthotospovirus* (TSWV). **(D)** Necrotic zonate spot in tomato infected with tomato necrotic spot-associated orthotospovirus (TNSaV). **(E)** Chlorotic spot in sweet pepper (*C. annuum* L.) infected with TZSV. **(F)** Necrotic ringspot in potato (*Solanum tuberosum* L.) infected with TZSV.

Symptoms of orthotospoviral disease in host plants are very similar, with only minor differences between species of virus. The major symptoms are ringspots (including chlorotic, yellow, necrotic, and zonate spots), bud necrosis, silver mottle, and vein banding. Zonate spots are characteristic of orthotospovirus infection. Although symptoms vary between disease stages, chlorotic, yellow, and necrotic ringspots can occur at all stages (early, middle, and late). Herbaceous plants with severe disease die in the late stage. The symptoms occur in the leaves and fruit, with a few cases of stem necrosis (Figure 1).

## Host Range

The host range of orthotospovirus has expanded from crops to other plants, including weeds such as *Bidens bipinnata* (Zhang et al., 2020) and even to woody plants such as kiwifruit, mulberry, and macadamia nut (Fang et al., 2013; Meng et al., 2015; Wang et al., 2016). Orthotospoviral diseases in China mainly involve diseases of vegetables, fruits, tobacco, groundnuts, and ornamental plants, including crops of the families Solanaceae, Cucurbitaceae, Asteraceae, Brassicaceae, Fabaceae, Orchidaceae, and Amaryllidaceae (Alliaceae), weeds (such as *B. bipinnata* L.), and woody plants (such as kiwifruit, macadamia nut, and mulberry) (Table 1).

**TABLE 1:** Orthotospoviruses reported by China.

Phylogenetic clade^*a*^	Species^*b*^	Abbreviation	Region	Host	Citations
WSMoV	Calla lily chlorotic spot orthotospovirus	CCSV	Taiwan, Zhejiang, Yunnan	Calla lilies (*Zantedeschia* sp.), Celtuce (*Lactuca sativa* var. *augustana*), Tobacco (*Nicotiana tabacum* L.)	Chen et al., 2005; Liu et al., 2012; Wu et al., 2018
WSMoV	Capsicum chlorosis orthotospovirus	CaCV	Taiwan, Sandong, Yunnan, Hubei, Guangdong	Phalaenopsis (*Phalaenopsis aphrodite Rchb.* F.), Calla lilies, Peanut (*Arachis hypogaea Linn.*), Tomato (*Lycopersicon esculentum* Mill.), Zucchini (*Cucurbita pepo* L.)	Chen et al., 2006, 2007, Chen et al., 2012a; Zheng et al., 2008; Huang et al., 2010; Yin et al., 2015; Sun et al., 2018
WSMoV	Mulberry vein banding orthotospovirus	MVBaV	Guangxi	Mulberry (*Morus alba* L.)	Meng et al., 2015.
WSMoV	Melon yellow spot orthotospovirus	MYSV	Taiwan, Guangdong, Hainan	Watermelon (*Citrullus lanatus* (Thunb.) Matsum. et Nakai), Cucumber (*Cucumis sativus* L.), Melon (*Cucumis melo* L.)	Chao et al., 2010; Chen et al., 2010; Liu et al., 2010; Peng et al., 2011
WSMoV	Pepper chlorotic spot orthotospovirus	PCSV	Taiwan, Yunnan	Sweet pepper (*Capsicum frutescens* L.), Chili pepper (*Capsicum annuum* L.)	Cheng et al., 2013; Zheng et al., 2017
WSMoV	Tomato necrotic spot-associated orthotospovirus	TNSaV	Guizhou	Tomato, Kiwifruit (*Actinidia* sp.)	Yin et al., 2014b; Wang et al., 2016; Zheng et al., 2016
WSMoV	Tomato zonate spot orthotospovirus	TZSV	Yunnan, Guangxi	Tomato, tabacco, Potato(*Solanum tuberosum* L.), Chili pepper, Iris tectorum (*Iris tectorum* Maxim.)	Dong et al., 2008; Cai et al., 2011; Li et al., 2014; Huang et al., 2015; Zheng et al., 2015a, c
WSMoV	Watermelon bud necrosis orthotospovirus	WBNV	Taiwan	Watermelon	Li et al., 2011
WSMoV	*Watermelon silver mottle orthotospovirus*	WSMoV	Taiwan, Yunnan, Guangdong,	Watermelon	Chu and Yeh, 1998; Chu et al., 2001; Rao et al., 2013; Yin et al., 2014a
WSMoV	Chili yellow ringspot orthotospovirus	CYRSV	Yunnan	Tomato, Chili Pepper	Zheng et al., 2020
TSWV	*Groundnut ringspot orthotospovirus*	GRSV	Yunnan	Potato	Ding et al., 2004
TSWV	*Impatiens necrotic spot orthotospovirus*	INSV	Taiwan, Yunnan	*Hippeastrum.*sp., Phalaenopsis, Dendrobium (*Dendrobium nobile*)	Adam et al., 1993; Zhang Q. et al., 2010
TSWV	*Tomato spotted wilt orthotospovirus*	TSWV	Yunnan, Taiwan, Beijing, Liaoning, Guangdong, Heilongjiang, Shaanxi, Ningxia, Sichuan, Qinghai, Shandong, Gansu, Guizhou, Tianjin, Chongqin, Hubei	Tomato, Watermelon, Peanut, *Chrysanthemum morifolium*, Chili pepper, Celtuce, *Allium sativum* L.Chinese, Parsley (*Petroselinum crispum*), Tobacco, Potato, *Pelargonium hortorum*, *Bidens bipinnata* L.	Adam et al., 1993; Zhang et al., 1998, 2017, 2020; Liu et al., 2010; Ren et al., 2014; Zheng et al., 2015b; Gao et al., 2016, 2020; Jie et al., 2017; Sun et al., 2017; Zhu et al., 2017; Song et al., 2019; Wang et al., 2019; Liu et al., 2019; Du et al., 2020; Jin et al., 2020; Wu et al., 2020; Zhang and Feng, 2020
IYSV	Hippeastrum chlorotic ringspot orthotospovirus	HCRV	Yunnan	*Hippeastrum* sp., *Zephyranthes candida*	Dong et al., 2013; Xu et al., 2013; Wu and Liu, 2017
IYSV	*Iris yellow spot orthotospovirus*	IYSV	Yunnan	Tobacoo	Wu and Liu, 2017
GYSV	Groundnut chlorotic fan-spot orthotospovirus	GCFSV	Taiwan,	Peanut	Chao et al., 2001
GYSV	*Groundnut yellow spot orthotospovirus*	GYSV	Yunnan	Chili pepper	Zhang Z. K. et al., 2010

*^a^List updated from clades defined by Peng et al. (2011) and Oliver and Whitfield (2016). ^b^Definitive species write in italics and tentative species write in upright letters.*

## Diversity and Evolutionary Relationships

**FIGURE 2:**
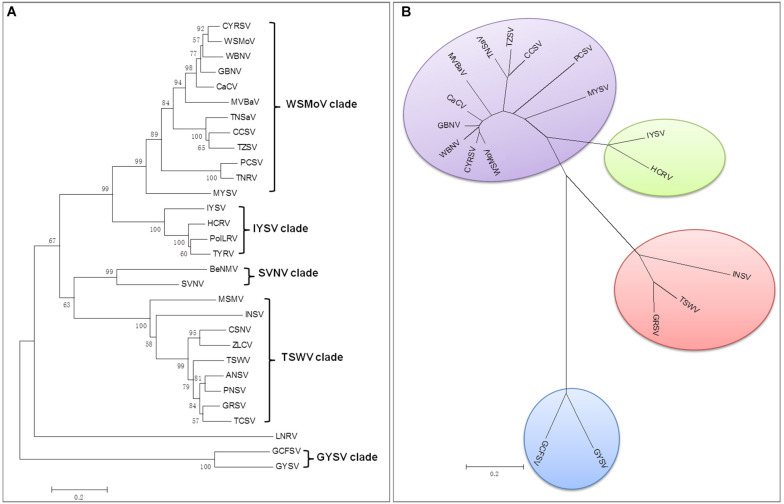
Phylogenetic tree of orthotospoviruses based on the amino acid sequence of nucleocapsid (N) protein. **(A)** Phylogenetic tree of 30 orthotospoviruses reported worldwide. **(B)** Phylogenetic tree of the 17 orthotospoviruses reported from China. Bootstrap values on the branches represent support for the branches based on 1,000 bootstrap replicates. Definitive species are written in italics and tentative species are written in upright letters. Abbreviations (and NCBI no.): ANSV, Alstroemeria necrotic spot orthotospovirus (GQ478668); BeNMV, Bean necrotic mosaic orthotospovirus (NC_018071); CaCV, Capsicum chlorosis orthotospovirus (NC_008301); CCSV, Calla lily chlorotic spot orthotospovirus (AY867502); CSNV, Chrysanthemum stem necrosis orthotospovirus (NC_027719); CYRSV, Chili yellow ringspot orthotospovirus (MH779495); GBNV, *Groundnut bud necrosis orthotospovirus* (NC_003619); GRSV, *Groundnut ringspot orthotospovirus*; GYSV, *Groundnut yellow spot orthotospovirus* (AF013994); HCRV, Hippeastrum chlorotic ringspot orthotospovirus (KC290943); INSV, *Impatiens necrotic spot orthotospovirus* (NC_003624); IYSV, *Iris yellow spot orthotospovirus* (AF001387); LNRV, Lisianthus necrotic ringspot orthotospovirus (AB852525); MSMV, Melon severe mosaic orthotospovirus (EU275149); MVBaV, Mulberry vein banding associated orthotospovirus (KM819701); MYSV, Melon yellow spot orthotospovirus (AB038343); GCFSV, Groundnut chlorotic fan-spot orthotospovirus (AF080526); PCSV, Pepper chlorotic spot orthotospovirus (KF383956); PolRSV, *Polygonum ringspot orthotospovirus* (KF383956); PNSV, Pepper necrotic spot orthotospovirus (HE584762); SVNV, Soybean vein necrosis-associated orthotospovirus (HQ728387); TCSV, *Tomato chlorotic spot orthotospovirus* (S54325); TSWV, *Tomato spotted wilt orthotospovirus* (NC_002051); TNRV, Tomato necrotic ringspot orthotospovirus (FJ489600); TNSaV, Tomato necrotic spot-associated orthotospovirus (KM355773); TYRV, Tomato yellow ring orthotospovirus (AY686718); TZSV, Tomato zonate spot orthotospovirus (NC_010489); WBNV, *Watermelon bud necrosis orthotospovirus* (EU249351); WSMoV, *Watermelon silver mottle orthotospovirus* (NC_003843); and ZLCV, *Zucchini lethal chlorosis orthotospovirus* (AF067069).

**FIGURE 3:**
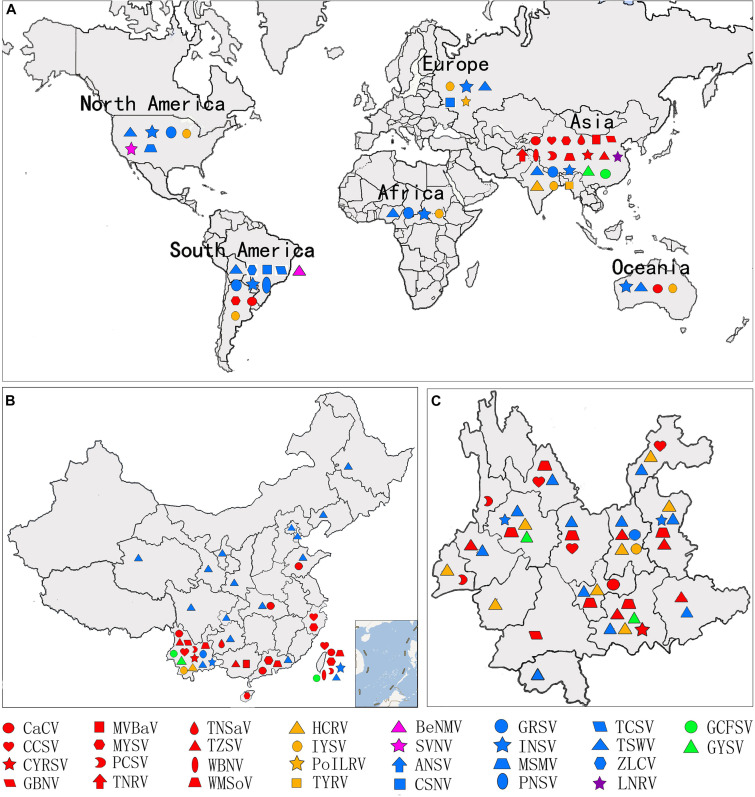
Geographic distribution of orthotospoviruses. **(A)** Worldwide geographic distribution of orthotospoviruses. **(B)** Geographic distribution of orthotospoviruses in China. **(C)** Geographic distribution of orthotospoviruses in Yunnan province, China.

There are currently 30 *Orthotospovirus* species known worldwide, comprising 11 definitive species (written in italics) and 19 tentative species (written in upright letters) (Figure 2A; International Committee on Taxonomy of Viruses Executive Committee [ICTVEC], 2020; Zheng et al., 2020). Orthotospoviruses can be divided into five phylogenetic clades based on the amino acid sequence of nucleocapsid (N) protein (Figure 2A), namely the *tomato spotted wilt orthotospovirus* (TSWV) clade, *watermelon silver mottle orthotospovirus* (WSMoV) clade, soybean vein necrosis-associated orthotospovirus (SVNV) clade, *iris yellow spot orthotospovirus* (IYSV) clade, and *groundnut yellow spot orthotospovirus* (GYSV) clade (Peng et al., 2011; Oliver and Whitfield, 2016). Based on the geography of the recent epidemics, viruses from the WSMoV and IYSV clades are mainly found in Asia and Europe (Figure 3A). Viruses from the WSMoV clade are most commonly found in East Asia and mainly infect crops in the families Solanaceae, Cucurbitaceae, and Asteraceae, with several new species from this clade reported in recent years (Dong et al., 2008; Yin et al., 2014b; Zheng et al., 2017, 2020). Viruses from the IYSV clade are mainly found in Central Asia and Europe. Some of the species in this clade, including IYSV and tomato yellow ring orthotospovirus (TYRV), were originally isolated from plants in the Middle East but have become endemic in Europe in recent years (Bag et al., 2015; Zarzynska-Nowak et al., 2016). Viruses in the GYSV clade, which is also Asian, have been reported from Taiwan province, China, and India (Satyanarayana et al., 1996; Chao et al., 2001). The SVNV and TSWV clades belong to the group found in the Americas. Soybean vein necrosis-associated orthotospovirus (SVNV) and bean necrotic mosaic orthotospovirus (BeNMV), two species in the SVNV clade, have been reported to infect bean plants in the United States and Brazil (Zhou et al., 2011; de Oliveira et al., 2012). Several members of the TSWV clade were originally found in the Americas (Torres et al., 2012; Webster et al., 2015). However, TSWV, which belongs to the TSWV clade, is now widely distributed throughout the world, and indeed is considered to be the most harmful of the *Orthotospovirus* species, causing great damage and large crop losses globally (Pappu, 2008; Pappu et al., 2009).

The country from which the largest number of *Orthotospovirus* species has been reported is China. A total of 17 *Orthotospovirus* species are known in China to date (Table 1), comprising 8 definitive and 10 tentative species. These species belong to four phylogenetic clades (TSWV, WSMoV, IYSV, and GYSV) (Figure 2B). Of the *Orthotospovirus* clades found in China, the WSMoV clade is the most diverse. Several new virus species in this clade have been reported for the first time in China (Dong et al., 2008; Yin et al., 2014b; Zheng et al., 2017, 2020), and it has been speculated that the WSMoV clade originated in China. TSWV, which belongs to the Americas group (Pappu et al., 2009), has become the virus posing the largest threat to agricultural production in China. Compared with native WSMoV clade viruses, TSWV has wider geographical adaptability (Figure 3B), and evidence of adaptive evolution of TSWV in China can be found in the phylogenetic analysis of TSWV *N* gene diversity (Mao et al., 2019; Liu et al., 2021).

## Occurrence and Geographical Distribution

Before 2000, only two *Orthotospovirus* species had been reported from China. The earliest record of TSWV symptoms in peanuts was published in 1986 by Xu in Guangdong and Guangxi provinces (Xu et al., 1986). Subsequently, TSWV-like virus particles were observed using transmission electron microscopy in tomato and tobacco from Sichuan and Yunnan provinces (Su et al., 1987; Yao, 1992; Zhang et al., 1998). WSMoV was first reported in Taiwan and identified as a new *Orthotospovirus* species through sequencing analysis (Chu and Yeh, 1998).

After 2000, the new *Orthotospovirus* species tomato zonate spot orthotospovirus (TZSV) was reported in tomatoes from Yunnan province, based on whole-genome sequencing and viral particle clustering characteristics in host cells (Dong et al., 2008). TZSV has a wide host range, causing harm in tomato, pepper, tobacco, and other crops, and it is now the dominant *Orthotospovirus* species found in these crops in Yunnan province (Huang et al., 2015; Zheng et al., 2015c).

Different *Orthotospovirus* species have been reported from different provinces, but TSWV is currently spreading rapidly from the south to the north of China. A total of 17 *Orthotospovirus* species have been reported from 19 Chinese provinces (Table 1 and Figure 3B). Yunnan has extremely high *Orthotospovirus* diversity, with 13 species identified, while most other Chinese provinces harbor only one or two *Orthotospovirus* species. Of all the *Orthotospoviruses*, TSWV has the most extensive distribution, occurring in 18 provinces to date (Table 1 and Figure 3B).

Yunnan has a high incidence of orthotospoviral disease, with diverse species of viruses and host plants, and a wide distribution. We identified 13 *Orthotospovirus* species from areas with very different climates (including tropical, subtropical, temperate, and cold temperate) (Figure 3C) and more than 20 species of natural host plants in Yunnan. The natural host range included most of the plants in China known to become infected with orthotospoviruses (Table 1). The orthotospoviruses isolated from Yunnan belonged to four phylogenetic clades, with the majority (seven species) belonging to the WSMoV clade. Three new *Orthotospovirus* species were first reported in Yunnan: TZSV (Dong et al., 2008), hippeastrum chlorotic ringspot orthotospovirus (HCRV; Dong et al., 2013), and chili yellow ringspot virus (CYRSV; Zheng et al., 2020). TSWV and TZSV were the dominant species, based on disease epidemics and severity (Zhang et al., 2016). The orthotospoviruses in Yunnan province have caused serious harm to crops including tomato, chili pepper, potato, and tobacco (Solanaceae); zucchini, watermelon, and squash (Cucurbitaceae); Phalaenopsis and Dendrobium (Orchidaceae); lettuce and chrysanthemum (Asteraceae); soybean and groundnut (Fabaceae), and more than 10 weed species.

## Transmission

Orthotospoviruses are becoming more and more harmful due to their multiple transmission routes and wide host ranges. The main transmission routes under natural conditions are via the vector thrips, which are insects in the order *Thysanoptera.* More than 14 species of thrips are known to act as vectors for orthotospoviruses.

The thrips that are known to transmit orthotospovirus include *Ceratothrip oidesclaratris*, *Dictyothrips betae*, *Frankliniella occidentalis*, *Frankliniella schultzei*, *Frankliniella gemina*, *Frankliniella intonosa*, *Frankliniella cephalica*, *Frankliniella bispinosa*, *Frankliniella fusca*, *Frankliniella zucchini*, *Scirtothrips dorsalis*, *Neohydatothrips variabilis, Thrips palmi*, and *Thrips tabaci* (Pappu et al., 2009; Whitfield et al., 2015). A single *Orthotospovirus* species can be transmitted by one or many species of thrips (e.g., TSWV is transmitted by nine species of thrips). A single species of thrips can transmit one or many *Orthotospovirus* species (e.g., *F. occidentalis* can transmit five *Orthotospovirus* species) (Whitfield et al., 2015).

Besides transmission by thrips, other routes of transmission facilitate spread of orthotospoviruses. Orthotospoviruses such as SVNV spread through seeds (Groves et al., 2016). In addition, spreading seedings infected with orthotospoviruses is also an important source for the virus to spread in different places (Zhang et al., 2020). Weeds, as an important primary infection source, provide potential conditions for the secondary infection and outbreak of orthotospoviruses in the field.

## Epidemiology

Orthotospoviruses have spread rapidly through China in recent years. Before 2015, orthotospoviral diseases mainly occurred in Southwest and Southeast China. However, over the last 5 years, orthotospoviral diseases have also occurred in Central, Northwest, and Northeast China. Although TSWV is the main epidemical *Orthotospovirus* species, we have recently found WSMoV, TZSV, and HCRV in Tibet, Hainan, and other Chinese provinces. Orthotospoviruses tend to quickly replace other viral pathogens (such as tobacco mosaic virus and cucumber mosaic virus) or form complex/mixed infections with other viruses (such as potato virus Y and whitefly transmitted geminivirus) in solanaceous crops, based on our research results over the past 20 years in Yunnan province.

The main reasons for the rapid expansion of *Orthotospovirus* species, especially TSWV, in China are as follows: (1) Over the last 20 years, the rapid popularization and development of greenhouses in China has provided perfect conditions for the propagation of the vector insects, thrips. Particularly in the north of China, greenhouses provide suitable temperatures for the overwintering of the virus-transmitting thrips, and indeed the orthotospoviral diseases in most northern provinces occur mainly in greenhouses (Jie et al., 2017; Liu et al., 2021). In southern China, as well as the greenhouse-thrips-vegetables infection pathway, a secondary infection cycle can be formed by weeds and other intermediate hosts, and weeds also provide thrips with overwintering habitats (Zhang et al., 2020). (2) Changes in planting structure have also led to the rapid expansion of TSWV. With the popularization of greenhouses, monocultures of a single crop variety with susceptibility to orthotospoviruses, such as pepper, tomato, and other Solanaceous crops, have been planted in large quantities (Miao, 2018). This is particularly obvious in Yunnan province, where the planting structure, which usually includes flowers, tomatoes, peppers, tobacco, and potatoes, provides very favorable conditions for the reproduction of the thrips population and susceptibility to orthotospoviruses (Zhao et al., 2021).

## Histopathological Aspects

Orthotospoviruses in host cells have distinct histopathological characteristics that mainly relate to the virus species rather than to the host plant species, and these characteristics have important diagnostic value. The histopathological characteristics differ between the different stages of infection. In general, at 3 days post inoculation (dpi), there are a large number of vesicles in the host cells. At 7 dpi, virus particles form in the vesicles. At this stage (in the early stages of infection), double-enveloped virions (DEV) are also observed in host cells. At 9 dpi, virus particle aggregates appear in the host cells. By 12 dpi (in the late stages of infection), the cells are filled with virus particles (Zhang, 2015).

**FIGURE 4:**
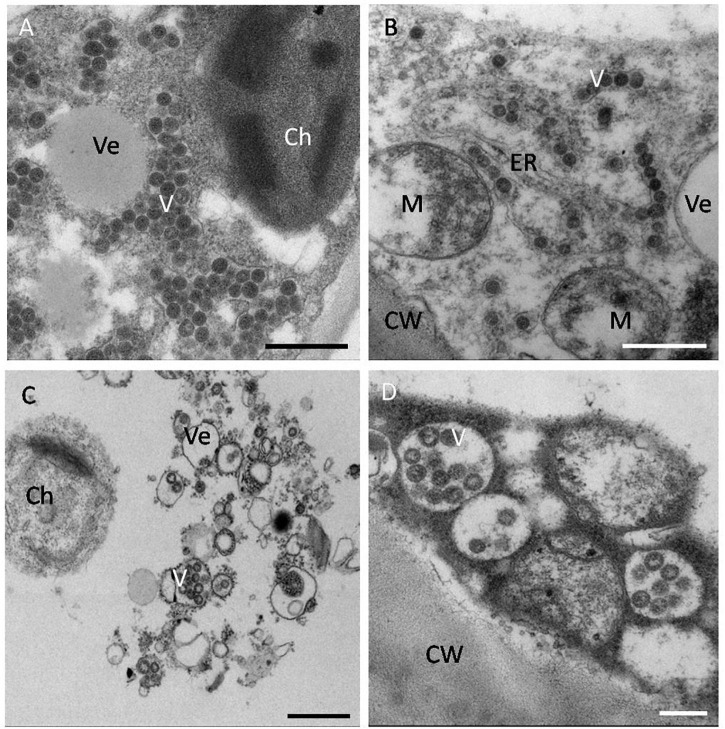
Histopathological characteristics of orthotospoviruses in host cells. **(A)** TSWV virus particles clustered in the cytoplasm of a tomato leaf cell. Bar = 500 nm. **(B)** TZSV virus particles clustered in the endoplasmic reticulum (ER) membrane of a tomato fruit cell. Bar = 500 nm. **(C)** WSMoV virus particles clustered in vesicles in a tomato fruit cell. Bar = 500 nm. **(D)** INSV virus particles clustered in the lumen of the ER in a tomato fruit cell. Bar = 20 nm. Ve, vesicle; V, virus particle; Ch, chloroplast; M, mitochondrion; ER, endoplasmic reticulum; CW, cell wall.

The virus particle clustering characteristics differ among *Orthotospovirus* species. TSWV virus particles always cluster in the endoplasmic reticulum (ER) or in special vesicles (based on transmission electron microscopy of ultrathin sections or negative staining). TZSV virus particles usually cluster in a moniliform structure in the ER membrane (vertical section) or as double-enveloped virions (cross-section) (Zhang et al., 2016). WSMoV virus particles always cluster in vesicles connected to other empty vesicles or in host cell vacuoles (unpublished data). *Impatiens necrotic spot orthotospovirus* (INSV) virus particles usually cluster in the lumen of the ER (Zhang Z. K. et al., 2010; Figure 4). Although these virus particle clustering characteristics are not necessarily unique to these viruses in the host cells, histopathological ultrastructure characteristics can help to identify *Orthotospovirus* species.

### Systemic Infection

To establish a systemic infection in a host plant, the TSWV ribonucleoprotein (RNP) complex migrates along the ER membrane. The N protein wraps with TSWV RNAs to form the RNP complex, and these RNPs are subsequently driven along the ER membrane and actin by the action of myosin, after which the viral RNPs enter into the Golgi body, where they form mature virus particles (Feng et al., 2013; Ribeiro et al., 2013). To infect a new cell, the RNP complexes move to the plasmodesmata, traversing the plasmodesmata through the action of the viral movement protein NSm to reach a neighboring cell. This process involves the interaction of the N-terminal of the NSm protein with nucleocapsid protein N and assists the movement of the RNPs toward the plasmodesmata using the actin microfilament/ER transport system (Leastro et al., 2015; Tripathi et al., 2015; Feng et al., 2016). The viral movement protein NSm plays a decisive role in the intercellular and long-distance movement of viral RNPs. However, research also shows that nucleocapsid protein N plays a key role in long-distance movement, and it has been confirmed that both TSWV N and NSm are necessary for the long-distance movement of movement-deficient TMV (Lewandowski and Adkins, 2005; Zhang et al., 2011). Deletion mutations have demonstrated that NSm mediates virus intercellular movement and long-distance movement using different domains, suggesting that the mechanisms by which NSm mediates virus intercellular movement and long-distance movement are different (Li et al., 2009). Interestingly, TSWV and TZSV virus particles with vesicles have been also found in plant vascular tissue, suggesting that viral particles can also load/unload from vascular tissues and establish systematic infection through long-distance movement (Zhang, 2015; Wen et al., 2020).

## Management

### Biological Control

Biological control is an effective method to control thrips-borne orthotospovirus disease, which includes diversified prevention and control measures. These include a reasonable rotation or continuous replanting mode (Bokil et al., 2019); release of predatory mites, mirids, and other natural enemies to the greenhouse environment (Bouagga et al., 2020); and adding beneficial microorganisms to the soil environment to help plants enhance disease resistance (Beris et al., 2018; Bonanomi et al., 2020). In disease management, integrated pest management (IPM) has been proved to be more effective than chemical control (Rodríguez et al., 2019).

### Screening of Virus-Resistant Plant Varieties

Growing highly virus-resistant plant varieties can be an effective way to prevent and control viral diseases. TSWV-resistant tomato and pepper varieties have been bred by Bayer, Syngenta, and other companies. These varieties possess the *Sw-5* and *Tsw* resistance genes, respectively. After selection trials, virus-resistant plants were grown in areas of high TSWV occurrence in China. However, the resistance of these plants was poor, due to single-gene resistance combined with the existence of complex/mixed infections. To date, there are no varieties with high levels of comprehensive resistance.

### Transgenic Resistance Breeding

Transgene technology is a rapid method for plants to acquire resistance to orthotospoviruses. So far, transgenic tobacco with broad-spectrum resistance against four other serologically unrelated *Orthotospovirus* TSWV, GYSV, INSV, and GCFCV has been developed by transforming the conserved motifs of the *RdRp* gene of WSMoV through hagrobacterium-mediated transformation (Kung et al., 2012; Peng et al., 2014).

### Screening of Natural Anti-orthotospoviral Chemicals

Natural chemicals have attracted increasing attention and have become the most promising strategy in the defense against pathogenic infections. These naturally occurring chemicals are popular because they are environmentally friendly and leave little residue, they are highly identifiable, and they have low toxicity for the plant hosts. Because of the serious damage to agricultural crops caused by TSWV infection, natural products able to limit TSWV have attracted a great deal of interest. One potential natural derivative is atin-3-acetonyl-3-hydroxyoxindole (AHO), isolated from *Strobilanthescusia*, which is able to up-regulate PR-10 genes in the salicylic acid (SA) pathway, as well as up-regulate the levels of miRNAs (miR156, miR172f, miR172g, miR408a) that contribute to inhibiting TSWV infection (Chen et al., 2014, 2017). Actigard, imidacloprid, and *Bacillus amyloliquefaciens* strain MBI600 have also been found to induce the SA signaling pathway and to prevent TSWV infection (Csinos et al., 2001; McPherson et al., 2005; Beris et al., 2018). The terpenoid compound 3α-angeloyloxy-9β-hydroxy-ent-kaur-16-en-19-oic acid (AHK) has been isolated from *Wedeliatrilobata* and found to defend against TSWV activity and infection, with an inhibition rate of 62.4% in curative effects assays and 76.5% in protective effects assays, mainly through activation of the jasmonic acid (JA) signaling pathway and inhibition of *NSs*, *NSm*, and *RdRp* gene expression (Zhao et al., 2019). Tagitinin A is a sesquiterpene isolated from *Tithonia diversifolia* and was found to have even higher curative and protective effects against TSWV, with an inhibition rate of over 75%. Furthermore, the expression of the genes *NSs* and *NSm* was inhibited in inoculated and systemic leaves in the protective assay, with an inhibition rate of more than 85% in systemic leaves (Zhao et al., 2017, 2020). It is therefore possible that these natural products can be used as chemical elicitors to trigger systemic acquired resistance (SAR), stimulating natural plant immunity. There is a wide variety of potential applications for such chemicals in agriculture.

### The Use of Virus-Free Seeding

Viral infection at the seedling stage (a highly susceptible stage) is the main reason for disease outbreaks in the middle and late plant growth periods. To obtain virus-free seeds and effectively reduce the occurrence and loss caused by orthotospovirus diseases, seedlings should be grown in greenhouses with insect-proof netting or maximal barrier precautions, yellow or blue sticky plates should be used to attract and trap the vector insects (thrips), and the virus carriage rate of the seedlings should be regularly monitored. Comprehensive measures to reduce the vector insects (thrips) should also be applied.

## Concluding Remarks and Future Prospects

Orthotospoviruses are expected to further expand worldwide because of their wide host range, multiple transmission routes, and ability to adapt to diverse climates. Additionally, increases in agricultural trade have accelerated global transmission. In China, new species or strains with high pathogenicity (because of mutation, reassortment, and recombination within and/or among *Orthotospovirus* species) have increased the frequency of disease. Orthotospoviruses are therefore a threat to crop production because of their high pathogenicity and the complex/mixed infections involving different *Orthotospovirus* species or other viruses.

It is necessary to enhance the monitoring of orthotospovirus infections and set up early warning systems in high-incidence areas; to allow the rapid diagnosis of symptoms caused by *Orthotospovirus* species in host plants; to assess virus carriage rates in seeds, seedlings, thrips, and weeds; and to allow dynamic monitoring of thrips levels. A deeper understanding of the mechanisms of systemic infection, of the pathogenesis of complex/mixed infections involving the same or different *Orthotospovirus* species or other viruses, and of *Orthotospovirus* adaptation mechanisms to multiple climate types is required, as is the screening and breeding of virus-resistant plant varieties. Furthermore, to ensure environmentally friendly prevention and control of orthotospoviral diseases, highly effective biological antiviral agents should be developed, and several techniques should be popularized, including the use of virus-free seeds and standardized seedling propagation technology (such as building greenhouses with insect-proof nets, sterilizing tools before raising seedlings, controlling the number of insects by using yellow or blue sticky plates, and releasing predatory mites, mirids, or the other natural enemies of thrips, and monitoring of virus incidence on seedlings before transplantation).

## Author Contributions

ZZ conceived and designed the review and wrote most of the manuscript. KZ wrote the text on orthotospoviruses evolutionary relationships, conducted the statistical analyses shown in Figure 2 and Table 1, and revised the manuscript. LZ wrote the text on the screening of natural anti-orthotospoviral products. XS conducted the statistical analyses shown in Figure 3. XZ wrote the text on orthotospoviruses transmission by thrips. TW conducted the transmission electron microscopy procedures. All authors contributed to the article and approved the submitted version.

## Conflict of Interest

The authors declare that the research was conducted in the absence of any commercial or financial relationships that could be construed as a potential conflict of interest.

## Publisher’s Note

All claims expressed in this article are solely those of the authors and do not necessarily represent those of their affiliated organizations, or those of the publisher, the editors and the reviewers. Any product that may be evaluated in this article, or claim that may be made by its manufacturer, is not guaranteed or endorsed by the publisher.
